# Costs and consequences of the conflict over infant sleep

**DOI:** 10.1093/emph/eou012

**Published:** 2014-04-08

**Authors:** Jon F. Wilkins

**Affiliations:** Department of the Fundamental Interconnectedness of All Things, Ronin Institute, Montclair, NJ 07043, USA

**Keywords:** genetic gonflict, genomic imprinting, parent-offspring conflict, coevolution

In the accompanying paper [1], David Haig describes the conflict over the inter-birth interval (IBI) in humans. Evolutionary theory predicts a conflict between a mother (who favors a shorter IBI) and her breastfeeding infant (who favors a longer delay before the mother’s next child). Haig argues that selection on the infant to delay the next pregnancy is likely to have been strong over human history, and that waking to breastfeed at night represents a possible adaptation on the part of the infant, as it will tend to delay the mother’s resumption of ovulation. The argument is further supported by sleep patterns observed in infants with Angelman and Prader–Willi Syndromes, which reveal the extension of this conflict to imprinted genes within the offspring, where alleles favor a shorter IBI when maternally inherited and a longer IBI when paternally inherited.

But what are the actual consequences of this conflict for human health? The evolutionary model indicates an arms race between the mother and infant strategies. That arms race will escalate until it reaches some sort of resolution. The cause and nature of that resolution will determine the severity of the fitness and health consequences of the evolutionary conflict.

One possible resolution is an outright victory by one of the factions. For example, if a maternal trait were to arise that fixed the IBI, making it completely resistant to manipulation by the infant, the IBI would evolve to the maternal optimum, and the coevolutionary arms race would be diffused. The conflict would still exist, in the sense that genes in the infant would still favor increasing the interval, but mutations lengthening the interval would no longer be available to natural selection. In fact, we would expect the infant to lose any IBI-lengthening traits it had previously acquired. The purifying selection required to maintain those traits would disappear, and the traits would be lost, just as sight, and even eyes, have been lost in certain fish that live entirely in lightless caves [2].

The evidence for adaptations to IBI manipulation in mother and infant (and at imprinted loci) suggests that an outright victory has not occurred. In the absence of such a victory, the arms race will continue until the accelerating fitness costs of side effects outweigh the benefits of further escalation. There are two broad classes of costs that are likely to be associated with negative consequences for both fitness and health.

One class of costs is associated with increased fragility of the system. Generally, biological systems are characterized by robustness—the ability to maintain an optimal, or near-optimal, phenotype in the presence of environmental or genetic perturbations. Conflict can undermine this robustness, particularly if the antagonistic coevolution involves escalation of gene expression.

For one thing, increased expression correlates with increased expression variance. In some systems, this can lead to increased phenotypic variance, so that individuals are, on average farther from the optimal phenotype [3]. Increased expression will also lead to the trait’s being constructed from multiple genes of large and opposing effects. A loss-of-function mutation in any of these genes would then lead to a large phenotypic perturbation with substantial health consequences, whereas in the absence of conflict, the phenotypic consequences of mutations in those same genes would be less severe.

The other class of costs is associated with pleiotropy—the fact that genes, and mutations in those genes, typically affect more than one trait. A mutation may be selected for its effect on the trait that is the basis of the conflict, but that mutation also likely affects other traits. In general, we expect that these pleiotropic effects to be deleterious: conflict over one trait can actually drive other traits to be less adapted. Pleiotropy also makes it possible that the evolutionarily stable value for the trait that is the focus of the conflict will actually lie outside the range defined by the optima of the conflicting factions [4]. For example, the evolutionarily stable IBI might wind up longer (or shorter) than what is favored by either mother or infant.

The focus of the conflict described here is the time to the mother’s next pregnancy, but the mechanism employed by the infant involves disruption its own sleep. Although the precise role of sleep is not fully understood, there is little doubt that it is important to health in general, and to cognitive health and development in particular. In infants, sleep consolidation seems to play an important role in language development [5] and executive tasks involving impulse control [6], pointing to a role in cognitive development.

Thus, an infant might benefit from delaying the birth of the next sibling, but it will pursue the delay only up to the point where that benefit is outweighed by the cost that its sleep disruption imposes on its own development. From this perspective, not only is night waking not adaptive, it is a side effect of conflict that may be both deleterious (in terms of fitness) and harmful (in terms of health).

Whether via fragility, pleiotropy, or a combination of the two, conflict leads to antagonistic coevolution, which leads in turn to the accumulation of deleterious traits. This is not just a likely outcome, it is a necessary one, as the conflict will not resolve until the accumulating side effects become detrimental enough that further escalation does not pay.

It is important to remember in general that natural selection does not necessarily guarantee positive health outcomes. But when conflict is a central driving force, selection can actually actively create negative ones.

**Conflict of interest**: None declared.
